# Familial Von Hippel–Lindau Disease: A Case Series of Cerebral Hemangioblastomas with MRI, Histopathological, and Genetic Correlations

**DOI:** 10.3390/life15111649

**Published:** 2025-10-22

**Authors:** Claudiu Matei, Ioana Boeras, Dan Orga Dumitriu, Cosmin Mutu, Adriana Popescu, Mihai Gabriel Cucu, Alexandru Calotă-Dobrescu, Bogdan Fetica, Diter Atasie

**Affiliations:** 1 Department of Clinical Surgical, Faculty of Medicine, “Lucian Blaga” University of Sibiu, 550024 Sibiu, Romania; claudiu.matei@ulbsibiu.ro; 2 Medlife Polisano Hospital Sibiu, 550024 Sibiu, Romania; atasie.diter@ulbsibiu.ro; 3 Biology and Ecology Research Center, Faculty of Sciences, “Lucian Blaga” University of Sibiu, 550012 Sibiu, Romania; 4 Department of Clinical Medicine, Faculty of Medicine, “Lucian Blaga” University of Sibiu, 550024 Sibiu, Romania; dan.orgadumitriu@ulbsibiu.ro (D.O.D.); cosmin.mutu@ulbsibiu.ro (C.M.); adriana.popescu@ulbsibiu.ro (A.P.); 5 County Emergency Clinical Hospital Sibiu, 550025 Sibiu, Romania; 6 Laboratory of Human Genomics, University of Medicine and Pharmacy of Craiova, 200638 Craiova, Romania; mihai.cucu@umfcv.ro; 7 Regional Centre of Medical Genetics, Emergency Clinical County Hospital Craiova, 200642 Craiova, Romania; alexcalota.crgm@gmail.com; 8 PATHOS Molecular Pathology Laboratory, Oncological Institute “Prof. Dr. Ion Chiricuta” Cluj-Napoca, 400015 Cluj-Napoca, Romania; feticab@yahoo.com

**Keywords:** Von-Hippel Lindau, VHL, hemangioblastoma, genetic disease

## Abstract

Von Hippel-Lindau (VHL) is a rare genetic disorder caused by mutations in the VHL gene on chromosome 3. The disease is associated with increased incidence of neoplasia. The most common manifestations of the disease are hemangioblastomas of the CNS and spinal cord, followed by renal cell carcinomas (RCC), pancreatic tumors, pheocromocytomas, endolymphatic sac tumors, and broad ligament or epididymal cystadenomas. Due to low incidence of the disease, information about its manifestation and genetic makeup has been slow to be gathered. Herein, we present three patients suffering from VHL, all part of the same family: patient one is the father; patient two is the daughter; and patient three is the nephew of the father, cousin to the daughter. Patients and their samples were investigated by magnetic resonance imaging, immunohistochemistry and genetic testing. Results show a tumor process in the left cerebellar hemisphere of the first patient which was successfully removed. The second patient presents with cervical medullary hemangioblastoma which was also successfully removed. The third patient had a tumor formation located at the craniospinal junction, at the level of the posterior bulb which was also treated. Genetic analysis showed patients one and two presented mutations in the VHL gene, confirming the VHL diagnosis. While the cases presented here follow the general lines for VHL disease, patients are related to each other, present tumors of the nervous system and mutations in the VHL gene, their particularities of presentation and manifestation bring new insights into this rare genetic disease.

## 1. Introduction

Von Hippel–Lindau (VHL) disease (MIM# 193300) is a rare genetic autosomal dominant disease with an incidence of 1 in 36 000 live births. VHL disease is associated with an increased incidence of neoplasia. The clinical manifestations of VHL include hemangioblastomas of the CNS and spinal cord, renal cell carcinomas (RCC), pancreatic tumors, pheocromocytomas, endolymphatic sac tumors, and broad ligament or epididymal cystadenomas. In the CNS haemangioblastomas are primarily found in the posterior fossa (especially the cerebellum) (50–60%), followed by the spinal cord (40–50%), and the brain stem (10–20%), but they have also been found in the pituitary stalk (2–4%). Supratentorial haemangioblastomas are uncommon (<2–5%) and those of the optic nerve are extremely rare [1].

Hemangioblastomas are rare, histologically benign (WHO grade I) tumors of uncertain histogenesis developed by approximately 70% of VHL patients. These patients represent 30–40% of all hemangioblastoma cases, while the other 60–70% are classified as sporadic [2]. From a clinical perspective, hemangioblastomas typically follow a saltatory growth pattern, characterized by periods of quiescence interspersed with episodes of rapid tumor expansion, which complicates surveillance and treatment planning [3]. More than 90% of patients with VHL develop multiple hemangioblastomas over the course of their lifetime, highlighting the multifocal nature of the disease. Symptomatology depends on tumor location and may include cerebellar signs (ataxia, dysmetria), cranial nerve dysfunction, spinal cord compression, or progressive visual loss from retinal involvement [4,5,6]. While many hemangioblastomas remain asymptomatic for prolonged periods, their potential for sudden growth and local mass effect makes regular clinical and radiological monitoring essential in VHL management [7]. Patients diagnosed with VHL usually die due to metastatic RCC and CNS lesions, and even though clinical treatments have improved, the life expectancy with VHL remains low, between 40 and 52 years.

Diagnosis of VHL disease in an individual with no known VHL family history can be established based on either the presence of two or more CNS hemangioblastomas or one hemangioblastoma plus either a visceral tumor or an endolymphatic sac tumor. If family history includes a case of VHL, the presence of any of the VHL-related tumors mentioned above is sufficient for diagnosis [8].

Von Hippel–Lindau disease (VHL) is caused by germline mutations of the VHL gene. A unique feature of the germline mutations in the VHL gene is the fact that the presence of heterozygous versus homozygous alleles leads to distinct pathological consequences. The heterozygous germline mutations are generally considered to be inactivating mutations, which, upon somatic loss of the wild-type allele, support tumor growth. Individuals homozygous (or trans-heterozygous) for germline VHL mutations have all been associated with the congenital form of the hematological disease familial erythrocytosis, an autosomal recessive disorder [9].

The VHL gene (3p25.3) [10], is a relatively small gene, conserved throughout evolution, spanning a region of about 10 kilobases. The gene comprises only three exons [11,12] and encodes two protein products, a larger full-length protein of 30 kDa (p30, NM_000551.2) containing all the 213 amino acids and a shorter, 19 kDa protein (p19, NM_198156.1), which contains only 160 amino acids. The shorter protein results from an alternative translation initiated downstream from the first start codon [13,14,15].

Both the p30 and p19 isoforms were shown to suppress tumor formation in nude mice [9], the mechanism of tumor suppression being mediated through hypoxia-inducible factor (HIF-a) degradation. In the event of a second somatic mutation of the VHL gene, the increased HIF levels lead to oncogenesis by activation of several angiogenic and growth signaling pathways [16].

In this study we present a case series of three related patients diagnosed with Von Hippel–Lindau disease. Each patient presented with manifestations of hemangioblastoma, a hallmark feature of VHL disease. The report describes their clinical manifestations, therapeutic interventions, and subsequent outcomes. The diagnosis of two of the patients was also confirmed by genetic testing. All three patients underwent successful treatment and subsequently achieved favorable recovery.

## 2. Materials and Methods

**Figure 1:**
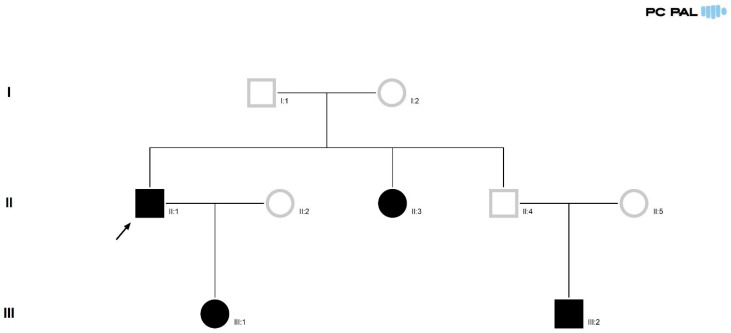
Pedigree of the three-generation family with 4 VHL positive members [12].

The study focuses on three patients suffering from VHL. Pedigree analysis of the relationship between the patients was done using PedigreeXP, (PC PAL, Bievres, France) (Figure 1) [17]. All patients were diagnosed and treated in the Polisano Hospital Sibiu, Sibiu County, Romania. Histopathologic processing and examination were carried out in the RaduSan Laboratory Cluj-Napoca, Cluj County, Romania.

### 2.1. Magnetic Resonance Imaging (MRI)

All MRI examinations were performed in the same imaging laboratory using a Siemens Aera 1.5T MRI scanner (Malvern, PA, USA). The cervical MRI examinations were performed using a standardized protocol applied across all imaging sessions. The protocol included sagittal T1-weighted, T2-weighted, and TIRM sequences, transverse (axial) T2-weighted sequences, as well as sagittal and transverse post-contrast sequences. The brain MRI protocol consisted of axial and coronal T2-weighted sequences, axial FLAIR sequences, diffusion-weighted imaging (DWI), susceptibility-weighted imaging (SWI), and native and post-contrast T1-weighted sequences. Multiplanar reconstructions were obtained from the post-contrast T1-weighted images.

Patients were analyzed by MRI both before and after surgical interventions. The analysis was done with both native and postcontrast IV. Different areas of the brain and spinal cord were analyzed depending on the complaint of the patient.

### 2.2. Histopathological Examination

Tumor samples were collected, embedded in paraffin, and stained with hematoxylin-eosin. Four samples were analyzed for patient one, three samples for patient two and one sample for patient three. Samples from patient two were also analyzed for detection of specific cellular and tumoral markers by immunohistochemistry with antibodies meant to detect the presence of CD34, S100, CD24, CKAE1/AE3, inhibin, GFAP, RP, SOX10, EMA, and Ki67.

### 2.3. Genetic Analysis

Whole-blood samples were collected on EDTA and shipped to the Regional Genomics Center in Craiova for processing. DNA was isolated and purified, following the manufacturer’s instructions, using Wizard^®^ Genomic DNA Purification Kit (Promega, Madison, WI, USA). Prior to the analysis for possible mutations in the VHL gene, DNA quantity and quality were assessed to ensure a minimum concentration of 20 ng/µL. The samples were further analyzed by multiplex ligation-dependent probe amplification (MLPA) using the SALSA MLPA kit (MRC-Holland, Amsterdam, The Netherlands) and specific probes targeting the genome region VHL 3p25.3 in order to diagnose the Von Hippel–Lindau syndrome, the amplification was performed on VeritiPro Thermal Cycler (Applied Biosystems, Waltham, MA, USA) and the capillary sequencing on a 3730xl DNA Analyzer (Applied Biosystems, Waltham, MA, USA). The specific probe mix used, P016-VHL (a semi-quantitative manual assay for the detection of deletions in the VHL and BRK1 genes in genomic DNA isolated from human peripheral whole blood specimens, with a precision of >99% as stated by the manufacturer), contains 29 probes targeting each VHL exon, genes located close to VHL, and references for detecting sequences on other chromosomes. The probe mix contains nine separate fragments to ensure quality control for the reactions. As a negative control, blood from the wife of patient one, and the mother of patient two, was also analyzed.

## 3. Results

### 3.1. Case Series

We present three patients, related to each other, who were diagnosed with Von–Hippel-Lindau syndrome; a 51-year-old male, suffering from recurring cerebral hemangioblastoma, first diagnosed at age 30 (case index—patient one), his daughter diagnosed with medullary hemangioblastoma (patient two), and his nephew diagnosed with bulbar hemangioblastoma (patient three). Another family member is affected, being diagnosed with pancreatic hemangioblastoma (Figure 1).

#### 3.1.1. Patient One

**Figure 2:**
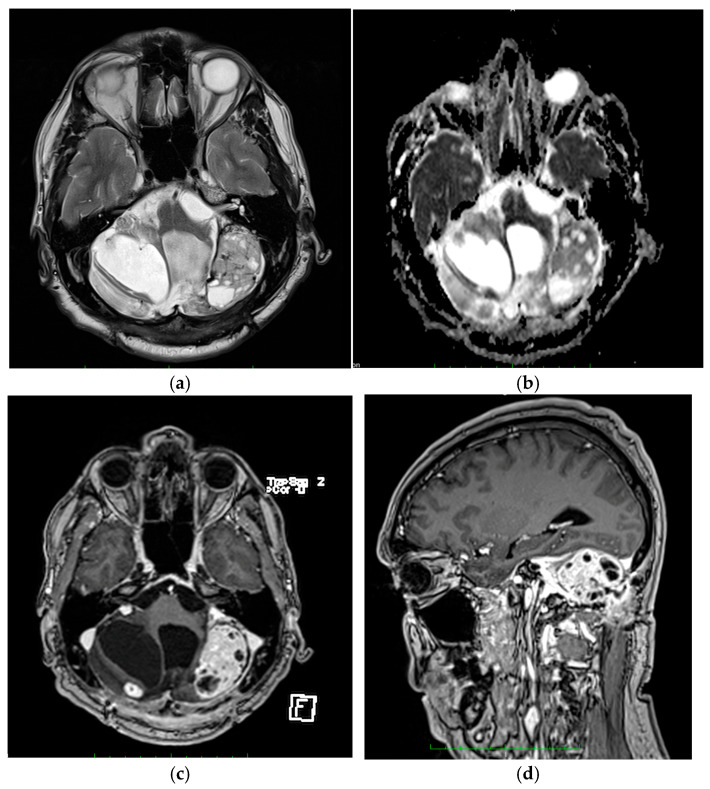
MRI results of patient one before surgery. (**a**) Image of an axial section though the T2 sequence, revealing a tumor process, which completely occupies the left cerebellar hemisphere, isointense, heterogeneous and with cystic areas inside. (**b**) Image of an axial section, diffusion sequence, which reveals the tumor process with marked restriction. (**c**) Image of an axial section, postcontrast T1 sequence, which highlights the tumor process located at the level of the left cerebellar hemisphere with intense and heterogeneous gadolinium contrast uptake; two more gadolinophilic nodules are present in the right pontocerebellar angle and right cerebellar convexity. (**d**) Image of a sagittal section, postcontrast T1 sequence.

Patient one was referred for the following symptoms: headache, dizziness, and inability to stand or walk. The patient had a clinical history of recurring CNS hemangioblastomas, with a previous surgery five years prior. Neurological examination showed the presence of intracranial hypertension, central vertigo, cerebellar ataxia, generalized hypotonia and hyporeflexia. Magnetic resonance imaging (MRI) examination indicated the presence of two tumor masses located at the level of the cerebellar hemispheres, one of 4 cm in diameter in the right hemisphere and the other of 5 cm in the left hemisphere (Figure 2). The tumors were hypointense in T1, hyperintense in T2 and FLAIR, restricted in diffusion and with a homogeneous and intense uptake of the contrast agent (Figure 2). No other abnormal findings were noted.

**Figure 3:**
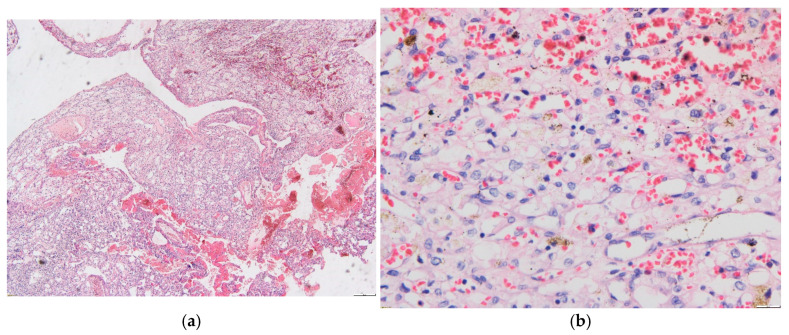
(**a**)—HE stain (40×) Nests and chords of stromal cells, numerous thin-walled vascular elements with mature endothelium, with occasional cystic and hemorrhagic areas. (**b**)—HE stain (400×) Compact proliferation of vascular structures and diffusely scattered stromal cells with vacuolated cytoplasm.

The patient underwent total tumor excision by occipital craniotomy, and samples were sent for histopathologic examination. The results indicated the presence of a tumor made up of nests and strands of stromal cells that exhibited abundant foamy or clear cytoplasm, mild nuclear pleomorphism, and numerous thin-walled vascular structures with mature endothelium. There were also occasional cystic or hemorrhagic areas and only a few mitotic figures, which is consistent with a diagnosis of hemangioblastoma (Figure 3).

**Figure 4:**
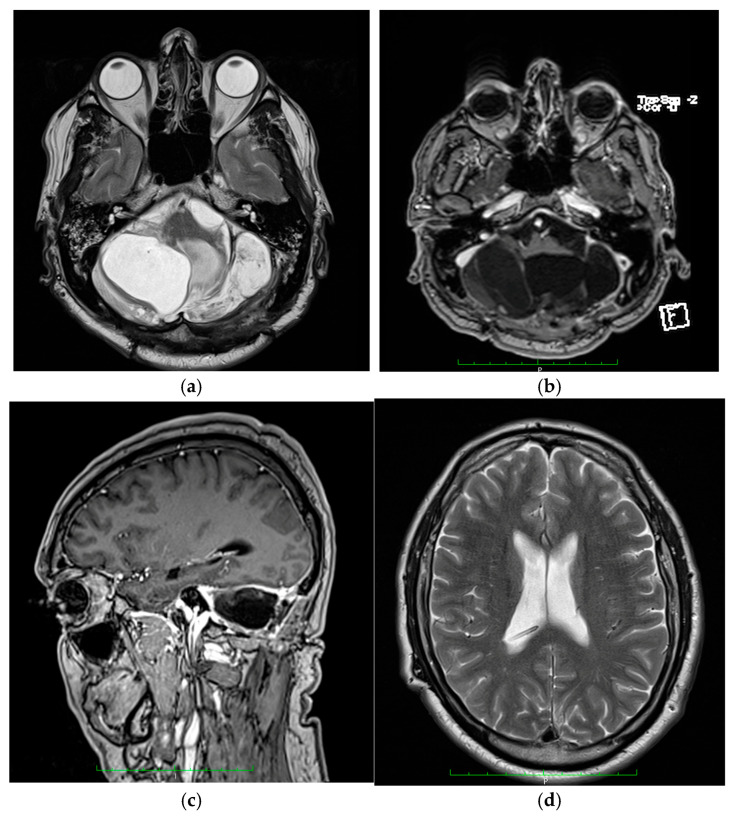
MRI results of patient one after surgery. (**a**) Axial section showing post-resection cystic cavity in the left cerebellar hemisphere. (**b**) Axial section, postcontrast T1 sequence, highlighting complete tumor resection. (**c**) Sagittal section, postcontrast T1 sequence. (**d**) Axial section, T2 sequence, which reveals a normal Taline ventricle system with a ventricular catheter placed at the level of the right ventricular atrium; the patient developed hydrocephalus therefore he was operated on and had a ventriculo-peritoneal drainage performed. All four frames were taken during the same MRI exam.

The patient underwent surgery and a suboccipital craniectomy was performed with total tumor resection and dura mater plasty. Nevertheless, the case was complicated by the occurrence of hydrocephalus, which was subsequently addressed through the implantation of a ventriculo-peritoneal drainage system. Figure 4 shows imaging frames from the postoperative MRI evaluation.

#### 3.1.2. Patient Two

**Figure 5:**
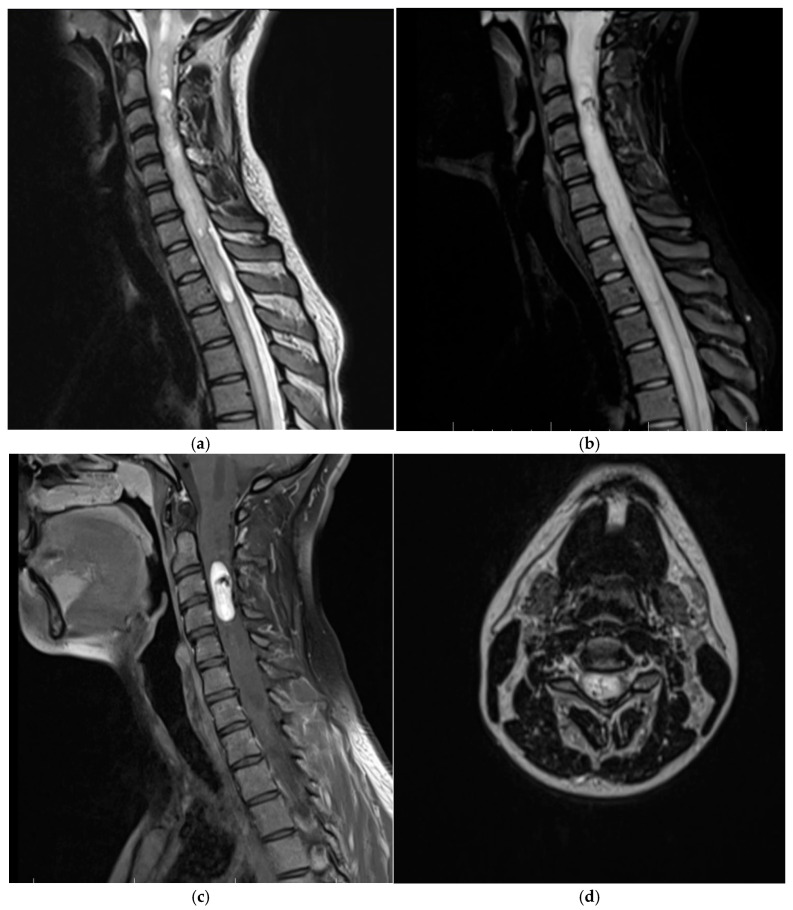
MRI preoperative exam of patient two revealing a cervical medullary hemangioblastoma. (**a**) Sagittal section, T2 sequence, which reveals a heterogeneous tumor process located intramedullary cervical, with isointense and hyperintense areas, having non-red flow void signals, due to dilated vessels, medullary edema and syringomyelia. (**b**) Sagittal section, T2 TIRM sequence, revealing marked perilesional marrow edema. (**c**) Sagittal section, postcontrast T1 sequence, which highlights an intramedullary tumor formation, located posterior to the C2 and C3 vertebral bodies, with intense and heterogeneous contrast uptake. (**d**) Axial section, postcontrast T1 sequence highlights the intermedullary location of the tumor.

Patient two, a 24-year-old female, the daughter of patient one, showed progressive loss of muscle strength with an insidious onset. The loss of muscle strength worsened approximately two weeks before the patient visited the clinic, at which point she lost the ability to stand or walk. The neurological examination noted mild cervical pain, spinal cord compression syndrome, spastic quadriplegia, reduced muscle strength (2/5 for the right arm, 3/5 for the right leg, 4/5 for both left limbs), clonic hyperreflexia, and bilateral positive Babinski sign. No sphincter disorders; gnosia, or lexia were present. The MRI examination of the entire craniospinal axis revealed a tumor formation located intramedullary at the C2-C3 cervical level. The tumor formation was hypointense in T1, hyperintense in T2 and FLAIR, with restriction in diffusion and an intense and homogeneous gadolinium intake, with a maximum diameter of 3 cm (Figure 5).

**Figure 6:**
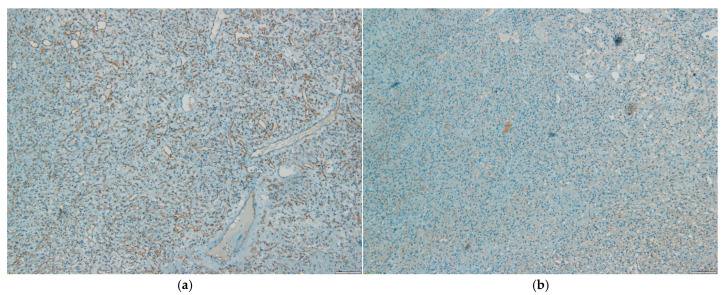
(**a**)—CD34 (100×): Vascular structures positive for CD34. (**b**)—S100 (100×): Stromal cells positive for S100.

The patient underwent total tumor excision by C2–C4 laminoplasty. Histopathologic examination revealed numerous small CD34 positive vascular structures and epithelioid stromal cells with clear cytoplasm, positive for S100 and negative for inhibin, CD24, CKAE1/AE3, GFAP, SOX10, PR and EMA (Figure 6). The diagnosis was hemangioblastoma.

After surgery the patient’s progress was very good, with clear improvement of the neurological deficits and healing of the surgical wound. The patient has improved significantly, being now fully independent and socio-professionally reintegrated, with no deficits and fully returned to normal life.

#### 3.1.3. Patient Three

Patient three is a 21-year-old male, the nephew of patient one, and cousin to patient two. The patient came to the clinic due to persistent hiccups, vomiting, paresthesia, and headache. The symptoms, which started 14 days prior, could not be treated with any of the following medications: Metoclopramide, Lidocaine, and Clonidine. The patient was able to walk and stand, and was diagnosed without Babinski and without sphyrian disorders.

**Figure 7:**
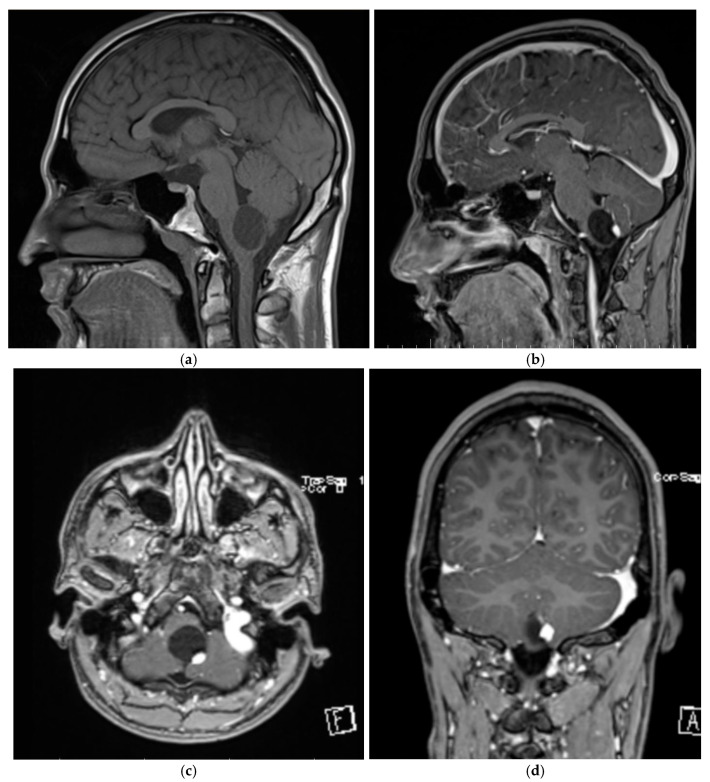
Preoperative brain MRI of patient three revealing a brain stem hemangioblastoma. (**a**) Sagittal section, native T1-weighted sequence, revealing a heterogeneous tumor process located at the craniospinal junction in the posterior part of the medulla oblongata, with cystic and solid, isopointense, well-defined composition. (**b**) Sagittal section, T1-T postcontrast sequence, revealing the intense and homogeneous gadolinium grip of the mural nodule. (**c**) Axial section, T1 sequence postcontrast. (**d**) Coronal section, T1 sequence postcontrast.

The brain and spinal MRI examination revealed a tumor formation located at the craniospinal junction, at the level of the posterior bulb. The tumor formation presented as a heterogenous structure composed of a 3 cm diameter cyst with a 1.2 cm solid mural nodule. It was hypointense in T1, hyperintense in T2 and FLAIR, with restriction in diffusion and an intense and homogeneous gadolinium intake (Figure 7).

**Figure 8:**
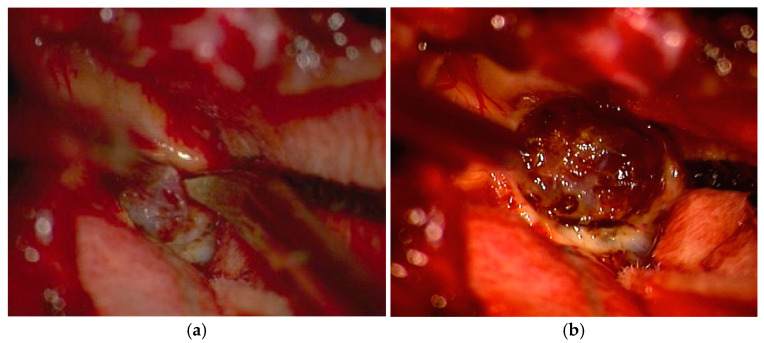
Intraoperative examination of patient 3. (**a**) The well-defined, red, mural nodule, intensely vascularized, with a diameter of 1.2 cm. (**b**) The tumor formation was completely removed.

The patient underwent total tumor excision by median suboccipital craniotomy and C1 posterior arch resection (Figure 8). The histopathologic examination showed a compact proliferation of vascular structures and diffusely scattered stromal cells with vacuolated cytoplasm, consistent with hemangioblastoma.

**Figure 9:**
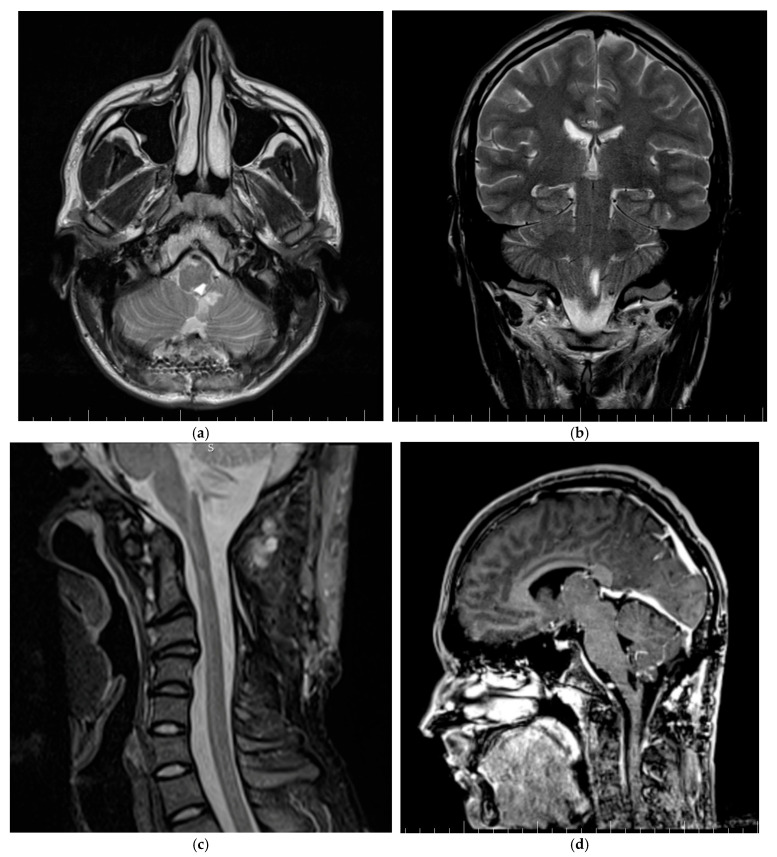
Postoperative MRI examination of patient three after complete excision of a brain stem hemangioblastoma. (**a**) Axial section, T2-weighted sequence, highlighting the complete remission of the cyst. (**b**) Coronal section, T2 sequence highlights the complete evacuation of the cyst and the presence of a post-resection cavity of the mural nodule. (**c**) sagittal section, TIRM sequence. (**d**) Sagittal section, T1 postcontrast sequence that highlights the complete resection of the mural node.

The postoperative evolution of patient three was favorable with clear neurological improvement and the disappearance of hiccups and other symptoms. The subsequent follow-up examinations confirmed the complete tumor resection, as well as the absence of complications (Figure 9). The patient is completely reintegrated socio-professionally, fully independent, with no deficits and fully returned to normal life.

### 3.2. Genetic Analysis

Of the three patients presented above, two consented to undergo genetic testing. Blood samples were collected for multiplex ligation-dependent probe amplification (MLPA) with the SALSA MLPA kit. The probe mix contains probes targeting each VHL exon, genes located close to VHL, and references for detecting sequences on other chromosomes specifically designed to diagnose the Von Hippel–Lindau syndrome.

Results of MLPA analyses show patient one and patient two present mutations in the VHL gene while the control patient does not. Patients one and two both present heterozygous deletions of all probes in the VHL gene (four probes for exon 1, three probes for exon 2 and two probes for exon 3), FANCD2 (two probes), BRK1 (one probe for exon 2 and one probe for exon 3), and IRAK2 (one probe). The control showed no deletion or duplication in the tested genes. Genetic analysis confirmed the clinical results.

## 4. Discussion

In this study, we report three cases of Von Hippel–Lindau (VHL)-associated hemangioblastomas occurring within a single family. Despite their close genetic relationship, the patients exhibited distinct tumor localizations: patient one developed a cerebellar hemangioblastoma, patient two a spinal cord hemagioblastoma, and patient three a brainstem lesion. None of the individuals demonstrated visceral manifestations of VHL, such as renal cell carcinoma, pheochromocytoma, or pancreatic tumors, which may be attributable to their relatively young age at diagnosis. Of particular note, patient two displayed an atypical immunohistochemical profile, with negativity for inhibin, CD24, CKAE1/AE3, GFAP, SOX10, PR, and EMA, and positivity restricted to S100. Genetic analysis using multiplex ligation-dependent probe amplification (MLPA) revealed heterozygous VHL mutations in two patients, corroborating the clinical and pathological diagnosis.

The patients in our case series were first diagnosed at ages 34, 19, and 21, consistent with the earlier onset of VHL associated hemangioblastomas compared with sporadic cases (mean age 29 vs. 47) [6,8]. Tumor site mirrors typical sites described in the literature: 45% cerebellum (patient one), 36% spinal cord (patient two), 11% cauda equina, and 7% brainstem (patient three) [2]. Interestingly, none of our cases showed associated visceral lesions typical of VHL, possibly due to the relatively young age of the patients, as the mean ages of onset for VHL associated renal cell carcinomas, pheocromocytomas or pancreatic tumors are 37, 30, and 36, respectively, with a large range of approximately ±30 years [8]. Although manifestations of VHL are varied throughout a patient’s lifetime they increase with age, involving more and more organs. The most dangerous visceral manifestation of VHL is renal cell carcinoma, which occurs in about 70% of affected individuals by age 60 years, and is a leading cause of mortality in VHL [18,19]. Presence of mutations in the BRK1 gene in patients one and two could explain why they have not developed renal cell carcinoma as these mutations have been associated with a reduced risk of renal cell carcinoma [20,21].

Another interesting finding is the atypical immunohistochemical profile of patient two, which was negative for inhibin, CD24, CKAE1/AE3, GFAP, SOX10, PR, and EMA, and positive for S100. Hemangioblastomas are usually positive for alpha-inhibin, D2-40, brachyury, NSE, NCAM1, S100, ezrin, CXCR4, aquaporin-1, and occasionally for GFAP and EGFR, and negative for EMA, CD10 and CAM5.2 [22].

Alpha-inhibin is a glycoproteic hormone involved in FSH secretion regulation, erythropoiesis, adrenal growth, retinal development and bone mass regulation [23]. Besides hemangioblastomas, it is usually expressed in adrenocortical tumors, sex cord tumors, and granulosa cell tumors [24]. Multiple studies suggest lack of inhibin expression is linked with tumorigenesis and unfavorable outcomes [24,25] but currently, it is not clinically used as a prognosis marker.

Although alpha-inhibin is used in IHC panels as a hemangioblastoma marker, it is known to be negative in rare cases. A PubMed literature review identified 10 case reports of inhibin negative hemangioblastomas (Table 1).

Although a majority of the cases presented in Table 1 feature hemangioblastomas with unusual locations, there is no correlation with inhibin negativity. A study of 57 cerebellar and 10 spinal hemangioblastomas found only one inhibin negative case [36]. Bukhari et al. reported four inhibin negative cases out of 20 spinal hemangioblastomas [37]. A study of 21 peripheral hemangioblastomas, 10 of which were confirmed or suspected to be VHL associated, found one negative case [38]. Muscarella et al. investigated 10 extraneuraxial cases, all of which were inhibin positive [39]. Hoang et al. reported no inhibin negative hemangioblastomas out of 25 cases, including 11 VHL related ones [40].

S100 positivity is consistent with hemangioblastoma, but non-specific. However, GFAP, SOX10, CD24, EMA, CKAE1/3, and PR negativity rule out meningiomas, glial tumors, schwannomas, metastatic melanomas or carcinomas [2,41]. Correlation with epidemiologic data, radiologic, macroscopic and H&E morphology strongly supports the diagnosis of hemangioblastoma.

It is well documented that VHL disease is caused by germline mutations of the VHL gene. Therefore, we tested the presence of these mutations as well as mutations in adjacent genes in two of the patients presented in this study. Both patients presented deletions in the VHL gene as well as in the other genes tested, supporting the clinical data and diagnosis [42]. Besides confirming the disease diagnosis these mutations can also shed light on clinical presentation and help in future management of the patients. Neither patient has, as yet, developed renal cell carcinoma. This could be explained by the presence of a mutation in the BRK1 gene which has been previously shown to confer protection to VHL patients from developing this type of tumor [21]. The large mutations identified in the two patients can also explain the earlier onset of disease in the daughter, at age 21, as compared to the father, at age 34 [43].

This study emphasizes some of the known features of VHL disease but also brings new data into focus. Familial aggregation is a well-recognized feature of VHL disease, and all patients in this series belong to the same family. However, although the individuals are closely related, they each present with different tumor sites highlighting intra-familial variability of VHL manifestations. Lack of visceral manifestations in either of the three patients may be related to their young age; however, it underlines the importance of long-term surveillance both for the patients and their close relatives. A novel and interesting finding of this study is the presence in patient two of a rare inhibin-negative hemangioblastoma with a restricted immunoprophyle (S100 only), adding to the small but growing body of literature on such cases. VHL is a genetic disease and detection of heterozygous VHL mutations via MLPA reinforces the diagnostic link between clinical, pathological, and molecular findings in this family cohort.

The main limitations of this study include the small sample size and the fact that all the patients belong to the same family. While the direct relationship between the patients is a common feature of VHL disease and provides valuable insights it also limits the generalizability of the findings as shared genetic and environmental factors may reduce variability in disease expression. Furthermore, two out of the three patients are relatively young and only recently diagnosed, therefore preventing a longitudinal assessment of the disease progression at this stage. Another limitation is the lack of an in-depth genetic analysis of all the patients and some of their relatives.

Future research should include continued monitoring of the current patients to establish longitudinal data and better characterize the progression of the disease over time. Also, the cohort could be expanded by recruiting additional family members who may have inherited the mutation and are therefore at risk. Data on new patients would provide valuable insight when studied in parallel with the already diagnosed cases. In addition, enrolling unrelated patients who present with similar clinical features could allow for comparative analyses between familial and sporadic cases. Finally, comprehensive genetic characterization of the current patients, including whole-genome sequencing through next-generation sequencing (NGS), would enable a more detailed assessment of VHL-associated and potentially novel genetic alterations.

## 5. Conclusions

Although rare, Von Hippel-Lindau disease and family screening should be taken into consideration when encountering hemangioblastomas, even in the absence of associated visceral lesions, as lack of these lesions could be explained by the genetic makeup of the patients. Moreover, early onset of disease can be expected in descendants of patients that present large deletions in the VHL and adjacent genes.

In rare cases, hemangioblastomas can present with atypical immunohistochemical profiles, underlining the importance of multidisciplinary communication between clinicians, radiologists and histopathologists in reaching the correct diagnosis. However, this should not affect the ability of clinicians to diagnose VHL in patients with hemangioblastoma, particularly in instances where they also have a family history of the disease.

Given the genetic and progressive nature of the disease, family screening and longitudinal observation are essential for early tumor detection in both patients and at-risk relatives.

## Figures and Tables

**Table 1:** Table presenting case reports of inhibiting negative hemangioblastomas found in the literature.

Case Report	Age/Sex	HB Site	Tumor Size	VHL	Immunoprofile, Inhibin (-) and:	
					Positive	Negative
Kaloostian et al., 2012 [26]	49/F	Supratentorial	6.5/5 cm	no	S100 NSE fXIIIa	GFAP EMA CD10 CAM5.2 RCCa
Rodrigues et al., 2013 [27]	56/F	Retina	ND	yes		S100 GFAP EMA CAM5.2 CD68
Basave et al., 2015 [28]	18/M	Gastric	5 cm	no	vimentin CD68	S100 GFAP EMA RCCa actin desmin synaptophysin HMB-45 CD117
Kasapas et al., 2020 [29]	36/M	Pituitary stalk	3 cm	no	NSE EGFR vimentin EMA	S100 GFAP CK5/6 CK7 CK20
Neal et al., 2021 [30]	51/F	Cervical, intramedullary (collision tumor with schwannoma)	1/0.5 cm	ND	S100 NSE	GFAP SOX10 CD68
Yang et al., 2021 [31]	12/M	Optic nerve (suprasellar)	2.35/1.85 cm	yes	NSE EGFR vimentin	S100 GFAP CD10 EMA CD56 CKpan Pax8 SSTR2
Li et al., 2022 [32]	33/F	Cerebellar hemisphere	3.9/3.4 and 2.1/1.5 cm	no	S100 D2-40 galectin-3 CA-9	GFAP EMA Olig2
Koo et al., 2022 [33]	54/F	Adrenal	4.2 cm	no	S100 NSE synaptophysin	EMA CD10 CD31 CA-9
Xu et al., 2022 [34]	51/M	Supratentorial	2.5/2 cm	ND	S100 PR	D2-40 GFAP CKpan EMA CD10
Kanehisa et al., 2022 [35]	66/F	Supratentorial	ND	no	D2-40	EMA
Our case (Patient 2)	24/F	Cervical, intramedullary	3 cm	yes	S100	GFAP SOX10 CD24 EMA CKAE1/3 PR

ND—not determined.

## Data Availability

The data described in this study are available upon request from the corresponding author.

## Abbreviations

The following abbreviations are used in this manuscript:

VHL	Von Hippel–Lindau
